# Screening of Bovine Coronavirus Multiepitope Vaccine Candidates: An Immunoinformatics Approach

**DOI:** 10.1155/2024/5986893

**Published:** 2024-07-18

**Authors:** Qian Jiang, Zhigang Ma, Fang Min, Xiaojun Ding, Yumeng Liang, Jinquan Wang, Lu Liu, Na Li, Yawei Sun, Qi Zhong, Gang Yao, Xuelian Ma

**Affiliations:** ^1^College of Veterinary Medicine, Xinjiang Agricultural University, Urumqi 830052, China; ^2^Xinjiang Key Laboratory of New Drug Study and Creation for Herbivorous Animal (XJ-KLNDSCHA), Xinjiang Agricultural University, Urumqi 830052, China; ^3^Institute of Animal Science, Xinjiang Academy of Animal Sciences, Urumqi 830011, China

## Abstract

Bovine coronavirus (BCoV) is a causative agent of enteric and respiratory disease in cattle. BCoV has been reported to cause a variety of animal diseases and is closely related to human coronaviruses; moreover, it has attracted extensive attention from both cattle farmers and researchers. With the rise of BCoV, a vaccine that is prophylactic and immunotherapeutic has to be utilized for a preemptive and adroit therapeutic approach. The aim of this study was to develop a novel multiepitope-based BCoV vaccine that can induce an immune response using a silicon reverse vaccinology approach. In this study, an immunoinformatics approach was employed to identify potential vaccine targets against BCoV, and four candidate antigen proteins were selected to predict B-cell and T-cell epitopes. To identify dominant epitopes, we employed a variety of bioinformatics techniques, including antigenicity prediction, immunogenicity assessment, allergenicity analysis, conservative analysis, and toxicity assessment. Finally, six multiepitope vaccines were developed using dominant epitopes, suitable adjuvants, Pan HLADR—binding epitope (PADRE), and linkers. Then based on the antigenicity score, solubility analysis, allergenicity evaluation, physicochemical property assessment, and tertiary structure verification score, construct 6 was selected as the best candidate vaccine; it was named CY. Molecular modeling and structural validation ensured the high-quality 3D structure of construct CY. The immunogenicity and complex stability of the vaccine were evaluated by molecular docking and kinetic simulation. In silicon clones, the BCoV vaccine had high levels of gene expression in the insect expression system. These results may contribute to the development of experimental BCoV vaccines with higher potency and safety.

## 1. Introduction

Bovine coronavirus (BCoV) is a single-stranded positive-sense RNA virus that belongs to the order Nidovirales, family Coronaviridae, subfamily Orthocoronavirinae, genus *Betacoronavirus*, and subgenus *Embecovirus* and is one of the major pathogens that causes diarrhea and respiratory diseases in calves [1]. The pathogen, which is mainly transmitted through fecal–oral infections and respiratory aerosols, causes diarrhea and respiratory symptoms and has been widely reported in the Americas, Europe, Asia, Oceania, and even Africa [2, 3, 4, 5, 6, 7, 8]. Geng et al. [9]conducted a systematic review and meta-analysis on the prevalence of BCoV in China before the year 2022 and revealed that BCoV is widespread in China [9]. In order to investigate the incidence of BCoV infection in Xinjiang, we conducted a 2-year follow-up survey of BCoV infection in Xinjiang from 2020 to 2022 on the prevalence of BCoV infection in the main producing areas of the cattle industry and further confirmed that there was also a high positive rate in Xinjiang [10]. The prevalence is as high as 8.2% in healthy cattle and as high as 79% in cattle exhibiting clinical signs [11, 12, 13, 14, 15]; the outbreak of this disease is difficult to control and causes significant economic losses to the cattle industry, which is characterized by intensive rearing.

In addition, the members of the *Betacoronavirus* genera include the emergence of the deadly human coronaviruses (HCoV), severe acute respiratory syndrome-related coronavirus (HCoV-SARS), Middle East respiratory syndrome-related coronavirus (MERS-CoV), and the severe acute respiratory syndrome coronavirus 2 (SARS-CoV-2), all of which are responsible for the current disastrous pandemics [16]. Among them, HCoV-OC43 is believed to be close to BCoV, the percentage sequence similarity between BCoV with HCoV-OC43 97% [17]. Moreover, the genome of coronavirus HECV-4408 isolated from children with acute diarrhea is more closely related to BCoV than other human coronaviruses, thereby indicating that this strain may be derived from BCoV, the percentage sequence similarity between BCoV with HECV-4408 over 99% [18]. Therefore, the risk of BCoV spreading to humans is extremely high and poses a great threat to public health [19].

Currently, there are two commercial vaccines available outside China that can be used to prevent and treat digestive tract diseases caused by BCoV infection in newborn calves. One is the inactivated vaccine Scour-Guard 3 (K), which is mainly used in cows in late pregnancy to increase the level of maternal antibodies acquired by newborn calves [20]. The other is a live attenuated calf vaccine called Calf-Guard (Pfizer Animal Health, NY), which is taken orally after birth to improve active immunity in calves [21]. However, clinical trials conducted by the Austrian Institute [22] confirmed that the immune effect of attenuated live vaccines and inactivated vaccines still has the potential risk of virulence atavism. Viruses may interfere with immunity and damage or change effective epitopes, thereby resulting in short maintenance time of the immune effect etc. There are also vaccination failures and safety issues due to poor host specificity or preference of viral vaccine strains and circulating wild strains. This suggests that these vaccines are inadequate and unsatisfactory in preventing BCoV infection.

Currently, there is no specific drug or effective method to prevent or control BCoV infection in China, and there is no commercially available BCoV vaccine. Vaccination and standardized prevention and control are one of the most effective ways to prevent BCoV infection [23]. Therefore, there is an urgent need to develop an effective and safe new vaccine to prevent the widespread epidemic of BCoV. This is also the main direction for future research.

Multiepitope vaccine combines immunogenomics with bioinformatics to search for new vaccine targets to design and develop multiepitope vaccines containing multiple virulence factors, stimulate the body to produce cellular and humoral immunity, and prevent viral diseases [23]. It is necessary to ensure that when disease vaccines are designed, the host immune response mechanism is effective and convenient. Compared with laboratory methods, the use of reliable virtual methods to screen highly conserved B-cell and T-cell epitopes with good immunogenicity to form a multiepitope antigen gene can reduce the cost, time, and risk of experiments [24, 25]. With the in-depth study on the molecular biology and immunology of viruses, it has been found that there are many T-/B-cell epitopes with strong conserved and immunogenicity on the antigens of these viruses, which can effectively cope with the variation of pathogenic microorganisms and some adverse factors in the immune response. Therefore, the immune response induced by epitope vaccines is highly targeted and safe [23]. In recent years, researchers have conducted predictive analyses of possible epitopes on related proteins of foot-and-mouth disease virus (FMDV) [26], avian influenza virus (AIV) [27], and mink enteritis virus (MEV) [28] and classical swine fever virus (CSFV) [29] synthesized recombinant multiepitope peptides, all of which produced high-titer neutralizing antibodies after immunizing experimental animals. The results revealed that the designed immunogen can stimulate the production of antibodies in vivo and the vaccine construction has good immunogenicity. The above research on epitope vaccines provides a new idea for the prevention of related viruses and has reference significance for the development of other effective multiepitope vaccines [23]. In summary, the multiepitope vaccine is feasible in preventing virus infection in animals and provides theoretical and experimental reference for the development of the BCoV multiepitope vaccine.

The purpose of this study was to construct a multiepitope vaccine candidate of BCoV. We used immunoinformatics approach to screen for four important pathogen structural proteins to design a multiepitope vaccine for the BCoV virus, in which the B-cell and T-cell epitopes used were obtained through immunoinformatics screening. The vaccine components also included link agents, adjuvants, and labels. We also analyzed the physicochemical properties, antigenicity, and sensitization of the multiepitope vaccine and performed molecular dynamics and molecular docking with TLR4, MHC I, and MHC II receptors. Finally, the vaccine was by silicon cloned. Overall, the vaccine showed satisfactory indicators in all analyses, but further validation is required from in vitro and in vivo trials.

## 2. Materials and Methods

**Figure 1:**
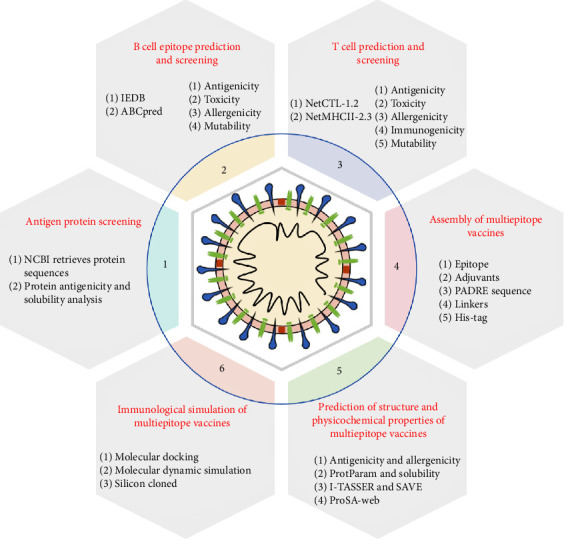
Flowchart of the design of BCoV multiepitope vaccine protocol using reverse vaccinology method.

In this study, a novel multiepitope vaccine was developed using immunoinformatics to prevent BCoV infection in cattle. The scheme in Figure 1 represents the entire approach for how the vaccine is ultimately constructed.

### 2.1. Collection and Screening of BCoV Protein Sequences

The whole proteome sequence of BCoV was collected from the National Center for Biotechnology Information (NCBI) Protein database (https://www.NCBI.nlm.nih.gov/genome), and its antigenicity was predicted [30, 31]. Each protein with the strongest antigenicity (>0.5) was selected for solubility prediction (>0.75) [32] and subcellular localization [33], and antigenic targets were screened for the preparation of the BCoV multiepitope vaccine.

### 2.2. Prediction of B-Cell (BCL) Epitopes

In this study, IEDB (https://BCellToolsiedb.org) and ABCPred (https://webs.iiitd.edu.in/cgibin/abcpred/test1_main.pl) were used to predict the linear B-cell epitopes of BCoV candidate antigenic proteins[34, 35]. Overlapping peptides from both predictions were selected for antigenicity (http://www.ddg-pharmfac.net/vaxijen/VaxiJen/VaxiJen.html), allergenicity (http://www.ddg-pharmfac.net/llerTOP/A), and toxicity (https://webs.iiitd.edu.in.raghava//ToxinPred/design.php) screening [32, 36, 37].

### 2.3. Prediction of Cytotoxic T-Cell (CTL) Epitopes

In this study, NetMHCpan-4.0 (https://services.healthtech.dtu.dk/services/NetMHCpan-4.0/) was used to predict the CTL epitopes of BCoV candidate antigenic proteins [38]. Epitopes with high sensitivity and specificity for bovine allele (BoLA) supertype were selected and entered into IEDB (https://ClassIImmunogenicityiedb.org) for immunogenicity prediction (>0) [34]. CTL epitopes with positive immunogenicity scores were screened, and the SYFPEITHI [39] database website was used (http://www.syfpeithi.de/bin/mhcserver.dll/) to enter the amino acid sequences of the above predicted CTL epitopes and select the CTL epitopes with higher scores (>12) for screening of antigenicity, allergenicity, and toxicity [31, 36, 37, 40].

### 2.4. Helper T-Lymphocyte (HTL) Epitope Prediction

We used NetMHCIIpan 4.3 [41] (https://services.healthtech.dtu.dk/services/NetMHCIIpan-4.3/) to preliminatively predict the HTL epitope of BCoV candidate antigen protein with a threshold of 0.5 and a strong binding site to the BoLA-DRB3 supertype with high sensitivity and specificity. The preliminary predicted HTL epitopes were entered into IEDB for immunogenicity prediction (>0) [34], HTL epitopes with positive immunogenicity scores were screened, and the abovementioned screened HTL epitopes were entered into the SYFPEITHI database website [39]. HTL epitopes with high scores (>12) were selected for antigenicity, allergenicity, and toxicity screening [31, 36, 37].

### 2.5. Conservative Analysis of B-Cell and T-Cell Epitopes

To check whether the region selected for antigenic epitopes faced any mutations, 98 HE protein sequences, 96 S protein sequences, 44 M protein sequences, and 94 N protein sequences were downloaded from the NCBI database and then compared using the MEGA server [42]. The results were viewed using the MView multiple alignment viewer [43]. Moreover, antigenic epitopes with high conservation were selected for the assembly of multiepitope vaccine constructs.

### 2.6. Assembly of the Multiepitope Vaccine

Six BCoV candidate multiepitope vaccines were constructed. Multiepitope vaccines contain an adjuvant, PADRE sequence, dominant CTL epitope, HTL epitope, and BCL epitope and utilize KK, AAY, and GPGPG linkers to connect the BCL epitope, CTL epitope, and HTL epitope, respectively [44, 45]. In the construction of the vaccine, three different adjuvant sequences were used: beta-defensin 3 (GenBank: AAV41025.1), heparin-bound hemagglutinin adhesion (HBHA) protein (from *Mycobacterium avium* subsp. paratuberculosis, GenBank: AGV15513.1), and L7/L12 ribosomal protein (*Mycobacterium tuberculosis*, GenBank: WP-003403353.1) [46].

There are two ways to assemble a multiepitope vaccine. One assembly method is to link BCL epitopes of all proteins, then the HTL epitopes of all proteins, and finally the CTL epitopes of all proteins (BCL-HTL-CTL). Another assembly method is to concatenate the epitopes of the HE protein, then the epitopes of the S protein, followed by the epitopes of the M protein, and finally the epitopes of the N protein (HE-S-M-N). In addition, after the addition of an adjuvant at the n-terminal, the connection is made via an EAAAK connector, which reduces interference with other protein fragments, thereby resulting in a more efficient separation. The addition of His-tags to the C-end is conducive to the purification of subsequent proteins [47]. After the linker, the insertion of a universal T-helper epitope Pan HLADR-binding epitope (PADRE) can significantly improve the antibody immune response induced by multiple recombinant antigen vaccines to further improve the immunogenicity of the vaccine [48].

### 2.7. Evaluation of Physicochemical Properties, Allergenicity, Antigenicity, and Solubility Evaluation of the Multiepitope Vaccine

The ExPASy server (https://web.expasy.org/protparam/) was used to evaluate the physicochemical properties of multiepitope vaccines, including isoelectric point (pI), molecular weight (MW), grand average of hydropathicity (GRAVY), aliphatic index, instability index, and half-life [49]. Using Vaxijen [31] online websites for predicting the antigenicity of multiepitope vaccine sequences, AllerTOP [36] was used to evaluate the allergenicity, of multiepitope vaccine sequences.

### 2.8. Secondary Structure Prediction and Tertiary Structure Refinement and Validation of Multiepitope Vaccines

The NetsrurfP-3.0 server was used to predict the secondary structure features of the multiepitope vaccine [50]. The tertiary structures of the vaccines were generated using the I-TASSER [51]. The most suitable model was selected based on its confidence score (*C*-score; typical range, −5, 2). GalaxyRefine was used to optimize the tertiary structure of the vaccine [52]. The optimized protein tertiary structure model was verified by two network servers ProSA and SAVE. The SAVE server (https://saves.mbi.ucla.edu/) to check the overall quality of the protein factors [53], and to create Ramachandran figure [54], forecasts the two aspects of amino acid residues body (psi (w) and phi (U)) [55], to determine after the elaboration of tertiary structure validity and rationality of the model. ProSA, a web server [56] (https://prosa.services.came.sbg.ac.at/prosa.php) to obtain the *Z* value, is used to check whether the input structure is characteristic of the natural protein in similar size fraction within the scope of the natural rationality of the evaluation model.

### 2.9. Conformational Prediction of the B-Cell Epitope

The online server ElliPro (http://iedb.org/ellipro/) was used to predict the conformational B-cell epitopes of the refined tertiary structure [57]. ElliPro provides a score for each output epitope, called the average PI value for each epitope residue. An ellipsoid with a PI value of 0.9 contains (90%) protein residues, while (10%) residues are outside the ellipsoid. The PI value of each epitope residue is determined based on the center of mass of the residue located outside the largest possible ellipsoid. Compared to other structure-based methods for predicting epitopes, ElliPro reaches the highest level and provides an AUC value (0.732) as the best calculation for any protein.

### 2.10. Analysis of Molecular Docking and Binding Capacity

To determine the extent of interaction and binding between multiepitope vaccines and bovine TLR4 (PDB ID: 3RG1), MHC I (PDB ID: 3PWV), and MHC II (PDB ID: 1SEB), molecular docking was performed using the HDOCK server [58]. Further, TLR4, MHC I, and MHC II target proteins were obtained from the RCSB database (https://www.rcsb.org/). HDOCK software was used to set each protein as rigid, the docking contact site as full surface, and the conformation generated after docking was set to 100 [54]. The docking score was calculated based on the knowledge iterative scoring function ITScorePP. A more negative docking score means a more likely bonding model. Finally the most negative docking is sorted into the best docking conformation for follow-up research and visualization. All protein structures were processed in the Molecular Operating Environment (MOE 2019.1) platform with the stand selected as Amber10, including the removal of water and ions, protonation, addition of missing atoms and complementation of missing groups, protein energy minimization, and optimization of the results for visual graphical analysis of the model using Pymol 2.1 software [59].

### 2.11. Molecular Dynamic Simulations

To further investigate the interaction of small molecules with proteins, we performed 100 ns molecular dynamic simulations of the protein–protein complexes using molecular dynamics [60]. The Gromacs 2020 software package was used to simulate the molecular dynamics of the screened receptor protein–small molecule complex. The AMBER99SB-ILDN force field parameter was used for protein, the TIP3P dominant water model was selected, the minimum distance of atoms in protein from the edge of the water box was 1.0 nm, and sodium ion or chloride ion was used to neutralize the system charge according to the docking result [54]. The root mean square deviation (RMSD), root mean square fluctuation (RMSF), radius of gyration (Rg) of the protein, solvent-accessible surface area (SASA), and the molecular dynamics of the protein–protein complex were obtained [59]. To gain more insight into the protein–protein binding, we counted the number of protein–protein hydrogen bonds throughout the simulation.

### 2.12. Codon Adaptation and In Silico Cloning

This study was conducted through an online website (https://www.vectorbuilder.cn/tool/codon-optimization.html) to predict the multiepitope vaccine codon adaptation index (CAI). The best expression system was selected by comparing the CAI—the higher the CAI, the higher the expression level of exogenous genes in the host [61]. SnapGene was used to clone the multiepitope vaccine on the appropriate expression vector [60].

## 3. Results

### 3.1. BCoV Protein Collection and Screening

All BCoV protein sequences were downloaded from the NCBI database. Sixty-four ORF1ab protein sequences, 45 NS2 protein sequences, 17 NS4.8 protein sequences, 98 HE protein sequences, 96 S protein sequences, 24 NS12.7 protein sequences, 57 E protein sequences, 44 M protein sequences, and 94 N protein sequences were used for antigenicity prediction. The strain with the strongest antigenicity of each protein was screened for antigenicity, and antigenic proteins with an antigen index greater than 0.5 (NS2, HE, S, NS12.7, M, and N) were screened for solubility prediction and subcellular localization (Table 1). Then, four proteins with the strongest solubility—HE protein (Protein ID QOV05127.1), S protein (Protein ID UZC49500.1), M protein (Protein ID AAY33957.1), and N protein (Protein ID QBH22433.1)—were selected for the preparation of multiepitope vaccines (Table 2).

### 3.2. Prediction of the BCL Epitope

The IEDB and ABCpred servers were used to predict BCL epitopes, and overlapping BCL epitopes with strong antigenicity (>0.5), no allergenicity, no toxicity, and no mutability were selected. The results revealed 12 BCL epitopes, including 3 BCL epitopes for HE protein, 5 BCL epitopes for S protein, 2 BCL epitopes for M protein, and 2 BCL epitopes for N protein (Table 3), which were used to create multiepitope vaccines.

### 3.3. Prediction of the CTL Epitope

CTL epitopes for four candidate proteins were predicted using the NetCTL 1.2 web server (9-mer). CTL epitopes with strong antigenicity (>0.5), immunogenicity (>0), no allergenicity, no toxicity, and no mutability were selected. The results revealed that there were 10 CTL antigen epitopes, including 3 CTL epitopes of HE protein, 4 CTL epitopes of S protein, 1 CTL epitope of M protein, and 2 CTL epitopes of N protein (Table 4).

### 3.4. Prediction of the HTL Epitope

HTL epitopes for four candidate proteins were predicted using the NetMHCII web server (15-mer). HTL epitopes with strong antigenicity (>0.5), immunogenicity (>0), no allergenicity, no toxicity, and no mutability were selected. The results revealed that there were six HTL antigen epitopes, including two HTL epitopes of HE protein, one HTL epitopes of S protein, one HTL epitope of M protein, and two HTL epitopes of N protein (Table 5).

### 3.5. Assembly of the Multiepitope Vaccine

**Figure 2:**
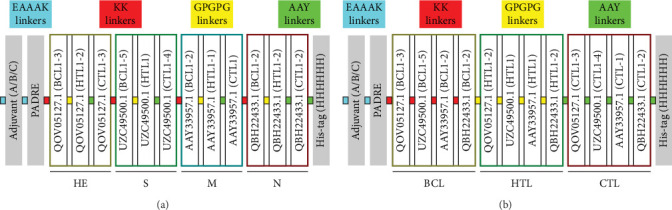
Design of multiepitope vaccine structure. (a) and (b) represent structural design of different permutations and combinations of multiepitope vaccines. BCL is linked by KK linkers in red, HTL is linked by GPGPG linkers in yellow, and CTL is linked by AAY linkers in green. At the N end, EAAAK linker connects the adjuvant and PADRE sequence in blue, and at the C end, AAY linker connects His-tag.

A total of 12 BCL epitopes, 10 CTL epitopes, and 6 HTL epitopes were used to construct the multiepitope vaccine. Six vaccines were designed, and each included a protein adjuvant (beta-defensin protein, HABA protein, and L7/L12 ribosomal protein), PADRE sequence, and T-cell and B-cell epitopes, as well as the linkers that linked them together (*Supplementary table 1*). The anticipated epitopes were separated by linkers (AAY, GPGPG, and KK). The EAAAK sequence was utilized to connect the initial adjuvant sequences to the PADRE sequence, thereby resulting in the production of the vaccine. The PADRE sequence was used to increase the potency and effectiveness of the peptide vaccines (Figure 2).

### 3.6. The Evaluation of the Allergenicity, Antigenicity, and Physicochemical Properties of Multiepitope Vaccines

The results showed that the structural instability index of 3 and 6 multiepitope vaccines was lower than 35, the antigen index was greater than 0.5, the half-life of each vaccine in *E. coli* (in vivo) was expected to be greater than 10 hr, and the hydrophilic value was greater than 0. The above two multiepitope vaccines have antigenicity, stable, hydrophilic, and no allergenicity proteins, which are consistent with the characteristics of vaccine candidate proteins (Table 6).

### 3.7. Prediction of Secondary Structure of Multiepitope Vaccines and Tertiary Structure Refinement and Validation of Multiepitope Vaccines

**Figure 3:**
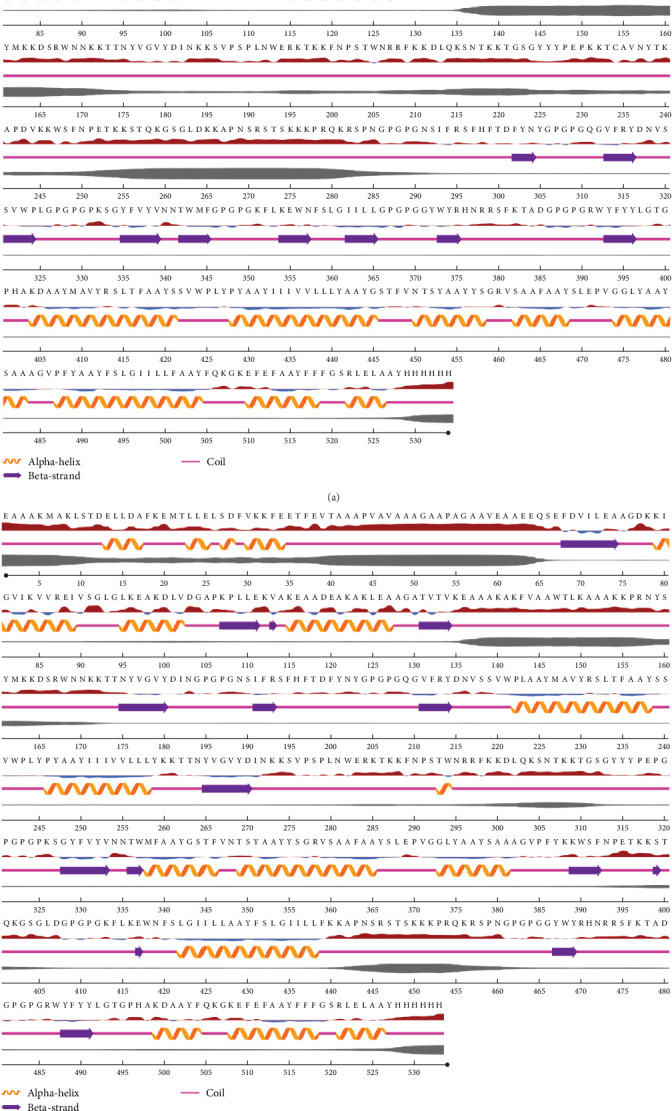
Secondary structure of multiepitope vaccines. *Note*. (a) Multiepitope vaccine 3 and (b) multiepitope vaccine 6.

The Protein-Sol online website was used to predict the secondary structure of the multiepitope vaccine. The results showed that the number of *α*-helix amino acids in the secondary structure of the multiepitope vaccine 3 was 128, accounting for 24%; the number of beta-strand amino acids was 52, accounting for 9.7%; and the number of coil amino acids was 354, accounting for 66.3%. The number of *α*-helix amino acids in the secondary structure of multiepitope vaccine 6 was 154, accounting for 28.9%; the number of beta-strand amino acids was 57, accounting for 10.7%; and the number of coil amino acids was 322, accounting for 60.4% (Figure 3).

**Figure 4:**
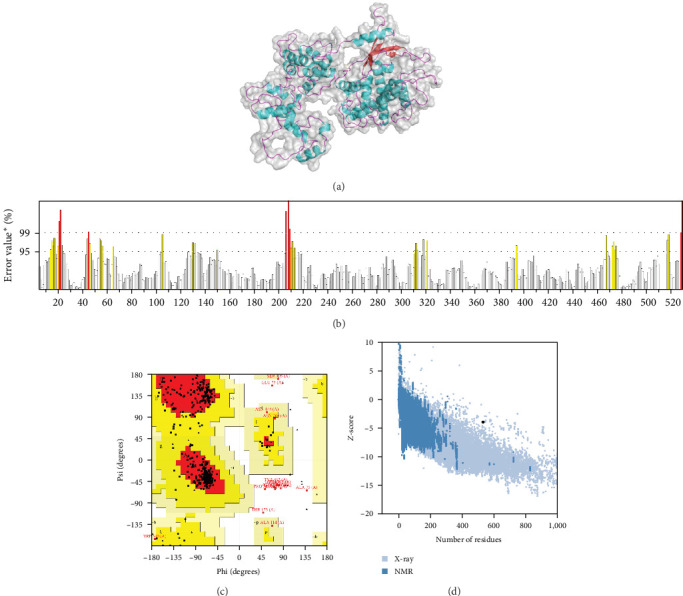
The refinement and validation of tertiary structure of six constructions. *Note*. (a) Six tertiary structure models; (b) ERRAT, with the overall quality factor of 92.233; (c) Ramachandran plot analysis showing 87.2% residues in the most favored regions, 9.5% residues in additional allowed regions, 0.9% residues in generously allowed regions, and 2.4% in disallowed regions of protein residues; and (d) ProSA-web, with a *Z*-score of−3.6. *⁣*^*∗*^, two lines indicate the confidence with which it is possible to reject regions that exceed that error value.

In addition, the I-TASSER server generated a tertiary structure model of the multiepitope vaccines 3 and 6 and received a *C*-score, which is the quality of the predicted tertiary structure model, usually in the range [2, 5]. The higher the *C-score*, the higher the confidence of the model. The results showed that the *C*-score of the tertiary structure of multiepitope vaccine 3 and 6 were −0.94 and −0.91, respectively. The Galaxy Refining server provides an optimized tertiary structure model for multiepitope vaccines 3 and 6. Then the SAVE server was used to evaluate the overall model quality and rationality of the optimized multiepitope vaccines 3 and 6 tertiary structure models. The higher the overall quality factor, the better the quality of the tertiary structure model. The overall quality factor of the optimized tertiary structure of multiepitope vaccines 3 and 6 were 89.544 and 92.233, respectively. According to Ramachandran's analysis, the ratio of amino acid residues in the most favorable allowable region of the tertiary structure of the optimized multiepitope vaccines 3 and 6 was 69.8% and 87.2%, respectively. The ProSA-web server was used to verify the rationality of the tertiary structure model optimized for multiepitope vaccines 3 and 6 according to *Z*-score. A low *Z*-score was considered to be a high-quality model with structure similar to that of natural proteins. The results showed that the tertiary structure *Z*-score optimized for multiepitope vaccine 3 and 6 were −2.08 and −3.6, respectively. Finally, 6 was selected as the best BCoV multiepitope vaccine and named CY (Figure 4 and *Supplementary figure 1*).

### 3.8. Conformational B-Cell Epitope Prediction

**Figure 5:**
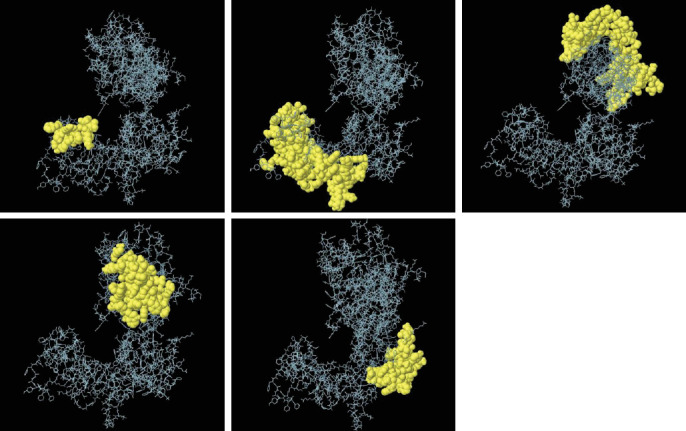
Three-dimensional representation of conformational B-cell epitopes of the designed multiepitope vaccine by ElliPro tool. *Note*. The yellow sphere represents the conformational B-cell epitope.

The conformational B-cell epitope of the multiepitope vaccine was predicted by Ellipro, and the conformational B-cell epitope with a score of 0.6 or above was selected. Of these five discontinuous B-cell epitopes, there are an estimated 270 residues with values ranging from 0.666 to 0.789 and conformational epitope residues with values ranging from 14 to 92 (Figure 5 and *Supplementary table 2*).

### 3.9. Analysis of Interaction between BCoV Multiepitope Vaccine and Receptor Protein

#### 3.9.1. Analysis of Interaction between CY Multiepitope Vaccine Protein and TLR4 Protein

**Figure 6:**
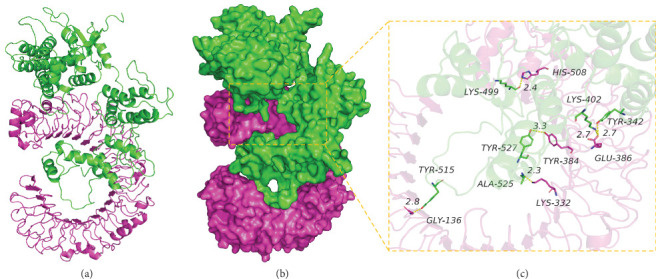
The binding mode of the complex CY with TLR4. (a) The backbone of protein was rendered in tube and colored in green (CY) and red (TLR4), (b) the CY and TLR4 protein is rendered by the surface, and (c) the detail binding mode of CY with TLR4. Yellow dash represents hydrogen bond.

The binding score of CY multiepitope vaccine protein and TLR4 protein was −375.49 kcal/mol. Further, the binding sites of CY protein included HIS-508, TYR-384, LYS-332, GLU-386, GLY-136, and other amino acid residues, and the binding sites of TLR4 included LYS-499, TYR-527, ALA-525, LYS-402, TYR-342, TYR-515, and other amino acid residues. CY and TLR4 protein contact residues can form a variety of interactions, such as hydrogen bonds (LYS-499, HIS-508; TYR-527, TYR-384; ALA-525, LYS-332; TYR-342, GLU-386; and TYR-515, GLY-136), salt bridges (LYS-402, GLU-386), and hydrophobic (TYR-527, TYR-384) and other interactions that can effectively improve the stability of CY and TLR4 protein complexes. In addition, according to the binding surface diagram of the two proteins, it was found that TLR4 protein and CY protein were well matched, which was conducive to creating a stable binding effect (Figure 6 and Table 7).

#### 3.9.2. Analysis of Interaction between CY Multiepitope Vaccine Protein and MHC I Protein

**Figure 7:**
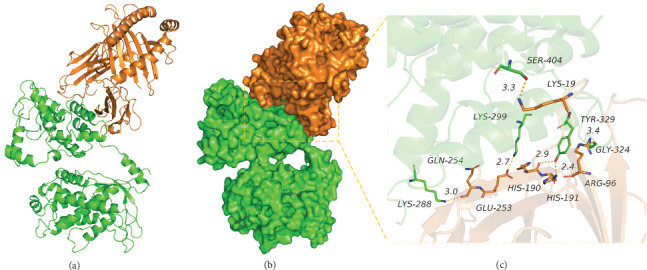
The binding mode of the complex CY with MHC I. (a) The backbone of protein was rendered in tube and colored in green (CY) and orange (MHC I), (b) the CY and MHC I protein is rendered by the surface, and (c) the detail binding mode of CY with MHC I. Yellow dash represents hydrogen bond.

The binding score of CY multiepitope vaccine protein and MHC I protein was 297.65 kcal/mol. Further, the binding sites of CY protein included LYS-288, LYS-299, TYR-329, GLY-324, SER-404, and other amino acid residues, and the binding sites of MHC I included A, GLN-254, GLU-253, HIS-190, and HIS-191, and B, ARG-96, LYS-19, and other amino acid residues. Moreover, CY and MHC I protein contact residues can form a variety of interactions, such as hydrogen bonds (GLN-254, LYS-288; HIS-190, TYR-329; HIS-191, TYR-329; ARG-96, GLY-324; and LYS-19, SER-404), salt bridges (GLU-253, LYS-299), and hydrophobic (ARG-96, TYR-329) and other interactions that can effectively improve the stability of CY and MHC I protein complexes. In addition, according to the binding surface maps of the two proteins, it was found that CY and MHC I proteins were well matched, which was conducive to forming a stable binding effect (Figure 7 and Table 7).

#### 3.9.3. Analysis of Interaction between CY Multiepitope Vaccine and MHC II Proteins

**Figure 8:**
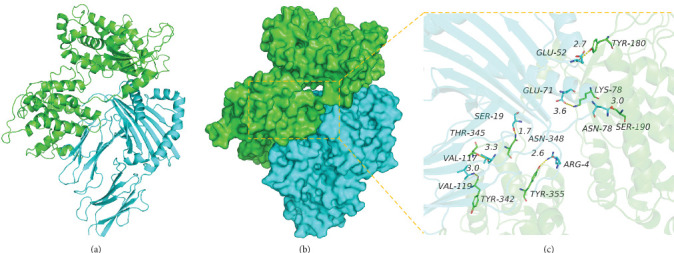
The binding mode of the complex CY with MHC II. (a) The backbone of protein was rendered in tube and colored in green (CY) and cyan (MHC II), (b) the CY and MHC II protein is rendered by the surface, and (c) the detail binding mode of CY with MHC II. Yellow dash represents hydrogen bond.

The binding score of the CY multiepitope vaccine and MHC II proteins was −294.57 kcal/mol. The binding sites of CY protein included amino acid residues, such as THR-345, TYR-342, ASN-348, SER-190, LYS-78, TYR-180, and TYR-355. The MHC II binding sites include A, VAL-117, VAL-119, SER-19, ASN-78, and GLU-71, and B, GLU-52, ARG-4, and other amino acid residues. CY and MHC II protein contact residues can form a variety of interactions, such as hydrogen bonds (VAL-117, THR-345; VAL-119, TYR-342; SER-19, ASN-348; ASN-78, SER-190; GLU-52, TYR-180; and ARG-4, TYR-355), salt bridges (GLU-71, LYS-78), and hydrophobic interactions (VAL-119, TYR-342), which can effectively improve the stability of CY and MHC II protein complexes. In addition, according to the binding surface maps of the two proteins, it was found that the MHC II protein and CY protein were well matched, which was conducive to creating a stable binding effect (Figure 8 and Table 7).

### 3.10. Molecular Dynamic Simulations Analysis

**Figure 9:**
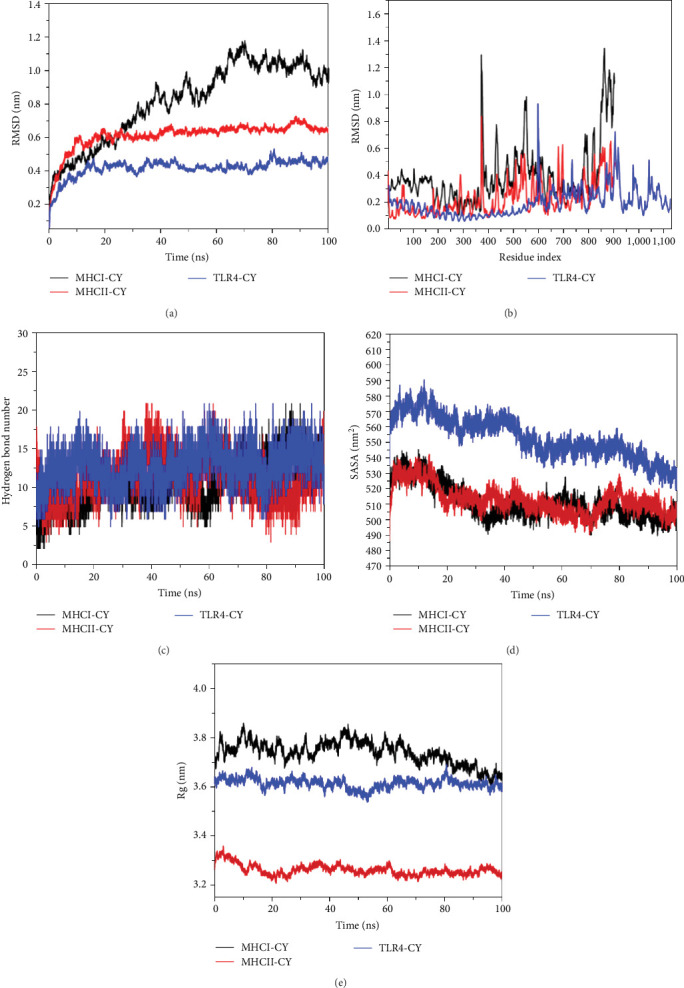
Molecular dynamic simulation of CY-TLR4, CY-MHC I, and CY-MHC II docking compounds. *Note*. (a) RMSD plot during molecular dynamic simulations for protein with protein complex. (b) RMSF plot during molecular dynamic simulations for protein complex. (c) The hydrogen bond number between protein and protein. (d) The surface area changes during the molecular dynamics. (e) The Rg changes of protein complex during the molecular dynamics.

It is evident from the RMSD plot (Figure 9(a)) that the average RMSD of the complexes are less than 0.65 nm, and the complexes basically attain dynamic equilibrium around 20 ns, which indicates that the proteins are well matched to each other and can form stable complexes. However, the CY-MHC I complex RMSD fluctuates slightly and reaches an equilibrium state at 70 ns. Importantly, we did not see any significant break in the RMSD curves, which also indicates that the two proteins are not separated and can be firmly bound to the protein to attain dynamic equilibrium. According to the RMSF plot displayed (Figure 9(b)), a small number of amino acid conformations in the complexes formed by protein–small molecule interactions undergo large changes (e.g., approximately 380, 550, and 860); however, most of them have small changes, which also reflects the stability of the complexes formed by protein–protein interactions, and this is the main reason for the small changes in the RMSD of the complexes. According to the Rg plot (Figure 9(c)), it is evident that the Rg of CY-TLR, CY-MCH I, and CY-MCH II proteins were reduced; in particular, that of the CY-MHC I complex was reduced rather significantly. According to the accessible surface area of all three complexes is significantly reduced (Figure 9(d)), which is mainly due to the enhanced interaction among the proteins, thereby resulting in a reduction in the polar area of the protein surface, which also indicates that the proteins bond tighter, which is beneficial in improving the stability of the proteins. According to the hydrogen bonding network diagram protein–protein interaction (Figure 9(e)), it is evident that after optimization by molecular dynamics, over 10 hydrogen bonding interactions are formed between protein amino acids, which play an important role in stabilizing protein–protein binding. Moreover, it also indicates that the binding between proteins is mainly driven by hydrogen bonding. The negative value of binding free energy (*Δ*Gbinding) highlights the stability of the system, while the positive value reveals the instability. Electrostatic interactions as well as van der Waals forces have a high performance in stabilizing proteins with proteins. The strongest binding was for the CY multiepitope vaccine protein and TLR4 protein with a low binding free energy (−9,018.477 ± 135.313 kJ/mol). The energy contribution of electrostatic interactions was −9,859.577 ± 104.444 kJ/mol, which was significantly higher than several other energy contributions, thereby indicating that hydrogen bonding in stabilizing protein–protein interactions plays an important role, which is consistent with the discussion on the number of hydrogen bonds. The binding between CY-MCH I and CY-MCH II proteins was also good, with binding free energies of −5,965.065 ± 131.567 kJ/mol and −6,413.673 ± 111.955 kJ/mol, respectively. Overall, there was strong hydrogen bonding between the proteins and a tendency for electrostatic interaction to drive binding (Table 8).

### 3.11. Codon Optimization and In Silico Cloning

**Figure 10:**
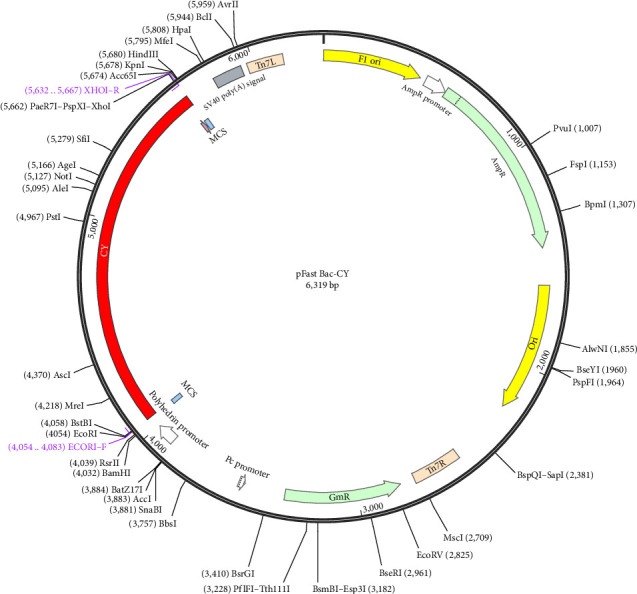
pFast Bac-CY recombinant expression plasmid. Using free SnapGene software (https://www.snapgene.com/free-trial/) and cloning epitope-based vaccine CY to pFast Bac1 expression vector, forming pFast Bac-CY recombinant plasmid. The red part represents the genetic code of the vaccine, and the black circle represents the backbone of the vector.

The results of predicting the codon adaptation index of the multiepitope vaccine showed a CAI of 0.63 in *Escherichia coli* (*E. coli*; strain, K-12), a CAI of 0.58 in *Pichia pastoris* (yeast), and a CAI of 0.86 in *Spodoptera frugiperda* (insect/SF9). The results indicated that the vaccine had a high expression in the insect expression system. The length of the optimized codon sequence was 1599 nucleotides, and the average GC content of the adapted sequence was 57.24%, with a CAI of 0.92. Finally, the adapted codon sequence was introduced into the plasmid vector pfast-Bac1 using SnapGene software to construct the recombinant plasmid sequence (Figure 10).

## 4. Discussion

BCoV is widely present in beef and dairy cattle and can cause diarrhea and respiratory disease in calves and adult cattle [1]. The disease occurs more often in the colder months of the year, usually near the calving season of cows. The incubation period of the disease is generally 2 to 8 days, and the main clinical characteristics are high incidence and mixed infection, generally 50%–100% [62]. The incidence of bovine coronavirus disease is high, but the mortality is low. Peng, AbiKeha, Yang, and Shen et al. conducted PCR detection on stool samples of diarrheal calves from various regions in China, and the results showed that the positive rate of BCoV was 17%–80.2%, indicating that BCoV is prevalent in cattle herds in most provinces in China to varying degrees [63, 64, 65, 66]. The widespread prevalence of BCoV not only caused great losses to the breeding industry but also seriously affected the growth and development of rehabilitated calves and the production performance of adult cattle. However, currently, there is no specific effective method to prevent or control BCoV infection in China, and there is no commercial BCoV vaccine [67]. Therefore, it is urgent to develop an effective and safe new vaccine to prevent the widespread epidemic of BCoV, which is also the main direction of future research.

Multiepitope vaccines are prepared based on the amino acid sequence of antigen epitope, which is a unique vaccine design idea developed in recent years and represents a new direction of vaccine design [8]. An effective vaccine should produce an effective humoral and cellular immune response to the target virus and infected cells, and the effectiveness of a vaccine depends largely on the selection of antigens [68]. Multiepitope vaccines are designed based on antigenic epitopes, which retain the pathogen proteins and help to combat the high mutability of RNA viruses [69]. Such vaccines focus on the protective immune response to pathogens and can eliminate adverse immune reactions [70]. It also has the advantages of safety, stability, cost-effectiveness, and time efficiency, thereby providing epitope options for designing better vaccine candidates [71]. For these reasons, our current study aimed to develop an epitope-based BCoV vaccine candidate using recognized silicon technology.

Because of the specificity of the immune response, the target epitope sequence directly determines the type of immune response. Given that both B-cell immunity and T-cell immunity are indispensable in BCoV clearance, the selection of appropriate immunogenic epitopes is a key step in BCoV vaccine design [72, 73]. The use of bioinformatics tools has shifted the direction of vaccine research toward the use of antigenic epitopes, which can stimulate immune responses in the body, thereby providing a better alternative to pathogen vaccines [74]. The focus of this study was to construct an epitope-based vaccine from BCoV structural proteins (HE, S, M, and N) using bioinformatics methods. These selected proteins have the potential to be effective vaccine candidates because they can trigger strong humoral and cell-mediated immune responses against HE, S, M, and N proteins during natural BCoV infection. Studies have revealed that neutralizing antibodies induced by S and HE proteins can significantly block the recognition pathway and reduce the infection ability of the virus [75]. N protein also contains multiple B-cell and T-cell epitopes, can induce strong protective antiviral immunity, has high immunogenicity, and is one of the potential targets of vaccines [76]. In addition, M protein plays an important role in viral assembly, and N protein binds to the hydrophilic amino acid at the carboxylated terminal of M protein, thereby influencing and determining the viral budding process [77]. All four proteins have been reported to be involved in immune escape strategies [78, 79, 80] and play a key role in the establishment of infection as effective candidate antigens against BCoV [81, 82, 83].

The target antigen in multiepitope vaccines carries epitopes that are presented by antigen-presenting cells to different T-cell subsets and further activated into helper T cells (Th cells) and cytotoxic T cells (Tc cells) [84]. The Th cells assist B cells in recognizing antigens and produce humoral immunity represented by antibodies, while Tc cells can directly recognize antigens and lyse-infected host cells to achieve cellular immunity [85, 86]. Therefore, the principle governing the prediction and screening of BCL, CTL, and HTL epitopes in this study was that they can induce strong humoral immunity and activate immunogenic cells to induce cellular immunity in the body [87]. Therefore, we preliminatively predicted BCL, CTL, and HTL epitopes on S, M, N, and HE proteins by using a variety of bioinformatics tools. However, not all predicted BCL, CTL, and HTL epitopes can be used to construct vaccines, and further screening is required. Allergenicity occurs when numerous vaccines drive the immune system [88]; in addition, the toxicity of epitopes can be harmful to the body. Moreover, the designed vaccine needs to be widely applied between different strains to effectively defend against BCoV infection. In conservative analysis, the epitopes are completely conserved (100%) between different virus strains, and only conserved sequences are used in the construction of multiepitope vaccines to provide a long-term defense response against infection with BCoV. Ultimately, we selected 12 BCL epitopes, 10 CTL epitopes, and 6 HTL epitopes, which were antigenic but had no sensitized potential to the host immune system and were nontoxic and conserved. The chimeric multiepitope vaccine generated by the selected epitopes may provide a good immune response.

In this study, the final multiepitope vaccine sequence was generated by arranging BCL, CTL, and HTL epitopes and linking them with a suitable linker, adjuvant, PADRE sequence, and His-tag. Researchers such as Bibi et al. [60], Uddin et al. [46], and Swetha et al. [89] have designed multiepitope vaccines that produce stronger immunogenicity by cleverly designing a vaccine structure that includes linkers and adjuvants. Therefore, in the process of vaccine design, it is rather important to select the appropriate adjuvant and linker. In this study, three different adjuvants—beta-defensin, HABA protein, and 50S ribosomal protein L7/L12—were selected and combined with two arrangements of BCL, CTL, and HTL epitopes, respectively. Six multiepitope vaccines against BCoV were designed. Among these adjuvants, beta-defensin displays a strong killing activity against pathogens, such as bacteria, viruses, and fungi; moreover, it can bind to different T-cell epitopes to induce different types of immune responses [90]. Another adjuvant is HBHA protein, an immunodominant antigen, a TLR4 agonist expressed that is known to induce a Th1-type response, stimulate T cells, and help produce IFN-*γ* production, thereby contributing to the development of effective immunotherapy strategies [91, 92]. Another adjuvant is the 50S L7/L12 ribosomal protein, which is actively involved in the translation process and is essential for elongation factor GTPase activity to improve immunogenicity at the cellular level [89, 93]. In addition to adjuvants, the rational use of linking agents can also improve immunogenicity. The PADRE sequence helps to activate CD^4+^ cells, and its ability to induce T cell response is more than a 1,000 times that of natural epitopes [94]. Therefore, we placed the PADRE sequence between adjuvant and BCL epitopes, further improving the ability to induce immune response in the body. An *α*-helix-forming ligand with an EAAAK sequence was added to the N-terminal of the adjuvant. As George and Heringa have proposed, many natural junctions exhibit *α*-helical structures [95]. The *α*-helical structure is more rigid and stable and includes hydrogen bond and mainchains, thereby maintaining a constant distance and independent function [96, 97]. Therefore, the rigid *α*-helix connector is considered to be a rigid spacer between adjuvants [98]. In this study, BCL epitopes were concatenated using KK ligands to avoid antibody induction into the middle of the two peptides, thereby helping to present each epitope specifically to the antibody [99]. Velders et al. confirmed that the amino acid sequence “AAY,” as a spacer peptide, its insertion into the epitope can improve the efficiency of the epitope cleavage [100]. HTL epitopes are also connected using GPGPG linkers, which—in addition to increasing the solubility of the construct—provide flexibility to adjacent domains with better epitope-presenting capabilities [101, 102, 103], thereby helping to stimulate T-cell helper responses and induce dependent immunogenicity. Based on the above principles, we integrated the EAAAK, KK, AAY, and GPGPG linkers between BCL, CTL, and HTL epitopes and connected the sequence of the adjuvant, PADRE, and His-tags labels and epitopes in a series to construct a multiepitope vaccine related to BCoV. The final structure reported in our study includes all the components required for a vaccine structure and also provides a better immune response.

A good vaccine candidate should have the ability to initiate an immune response without causing an allergic reaction. Therefore, we analyzed the antigenicity and sensitization of multiepitope vaccines. Except for V5, the antigen index was less than 0.5; the other five vaccines had strong antigenic and nonallergenic properties. Other properties revealed solubility, heat resistance, hydrophilicity, and stability. Except V5, the other five vaccine structures belonged to the good vaccine category. In the tertiary structure analysis, our V6 vaccine candidate had the highest *C*-score (−0.91) and overall quality factor of the five vaccine structures, thereby indicating that V6 had a higher quality three-dimensional structure than the other four vaccines. We finally used the galaxy refining and prevalidation server to refine and validate the 3D structure of the V6 vaccine structure, and with the optimization of the 3D structure of the protein, better properties were observed on the Ramachandran diagram. The results reveal that most of the residuals exist in the favorable region, and the residual in the outlier region is lower, which indicates that the quality of the designed model is satisfactory.

Conformational B-cell epitopes were predicted using the Ellipro tool of the IEDB server, with the majority of residues (188) having scores greater than 0.7 and only 82 residues having scores lower than 0.7. Therefore, we can infer that our vaccine builder V6 has an induction effect on B cells and can promote humoral immunity in the body.

Numerous promising vaccine constructions follow well-defined immune mechanisms that trigger the desired response in the host. These reactions are mainly due to the successful interaction of the vaccine construction with toll-like receptors (TLRs). When pathogens break through the mucosal barrier, TLRs can recognize them and initiate adaptive immune responses [104]. The TLR4 receptor is targeted and used in the development of an activated coronavirus vaccine because its activation promotes the production of inflammatory cytokines, thereby ensuring an effective immune response [105].

Further, MHCI and MHCII also play an important role in the immune response triggered by viral infection, with antigen-stimulated antigen-presenting cells (APCs) that can break down antigenic substances and assemble them with MHC molecules. Finally, the antigen–MHC complex is expressed on the cell surface, and this complex can interact with the T-cell receptor (TCR) on the T-cell surface and synergistically activate T cells with the interaction of helper molecules, thereby resulting in strong cellular immunity [106]. Therefore, we used the docking technology to study the binding affinity between V6 and different bovine immune receptors (TLR4, MHCI, and MHCII). The free binding energy calculated from the connection between V6 and immune receptors confirmed that the protein we developed may lead to a protective immune response, and the free energy of V6 binding to these three immune receptors was negative. The results reveal that the surface of the complex composed of immune receptors and multiepitope proteins is well matched, which can form a variety of interactions and produce stable binding effects in order to stimulate the body to produce an appropriate immune response.

We also performed molecular dynamic simulations of CY-TLR4, CY-MCH I, and CY-MCH II complexes using iMODS servers to evaluate their stability under atomic conditions. It is evident from the molecular dynamic simulation results that the constructed vaccine V6 can firmly bind to the three immune receptors and achieve a good dynamic balance. In order to evaluate the compactness of the three complexes, we calculated the Rg value, and the Rg of the three protein complexes decreased, which reflects that the binding of the proteins promoted more electrostatic and hydrophobic contacts to bind them tightly. In addition, in order to ensure the stability of the protein structure, we evaluated the hydrogen bonds; the constructed vaccine complex revealed the existence of sufficient hydrogen bonds, which indicated its stability. These findings indicate that the vaccine complex is stable and less fluid in the simulated environment. In summary, these three complexes perform well in molecular dynamic simulation studies.

The predictive framework for multiepitope vaccine design and its downstream analysis using molecular docking and interaction analysis are a good starting point for developing vaccine candidates. However, further in vitro and in vivo studies are required to determine whether the multiepitope vaccine constructed in this study provides protective immunity against BCoV infection. The next step in the current plan is to express the vaccine in the *S. frugiperda* (insect /SF9) system and to perform extensive immunological analyses to confirm the results obtained through immunoinformatics analysis.

## 5. Conclusion

In summary, we utilized a variety of immunoinformatics tools to develop a novel multiepitope BCoV vaccine with high immunogenicity. The results revealed that the vaccine had satisfactory antigenicity, solubility, nonsensitization, physical and chemical properties, and tertiary structure. However, only bioinformatics analysis is not enough. We will also confirm that BCoV multiepitope vaccine is an effective immunogenic candidate vaccine through immunization animal experiments, which provides a theoretical basis for the development of multiepitope vaccines.

## Figures and Tables

**Table 1:** Antigenicity prediction of the nine encoded proteins of BCoV.

Protein name	Protein ID	Length (Aa)	Antigenicity
NS2	USW46667.1	279	Antigen (0.741)
NS12.7	USW46671.1	102	Antigen (0.683)
N	QBH22433.1	448	Antigen (0.669)
HE	QOV05127.1	428	Antigen (0.611)
S	UZC49500.1	1,363	Antigen (0.567)
M	AAY33957.1	230	Antigen (0.538)
NS4.8	USW46670.1	29	Nonantigen (0.384)
E	AAY33950.1	84	Nonantigen (0.188)
ORF1ab	NP150073.1	7,094	Nonantigen (0.016)

*Note*. The mean antigenicity greater than 0.5 is considered as a strong antigenicity protein.

**Table 2:** Solubility prediction and subcellular localization of BCoV highly antigenic proteins.

Protein name	Protein ID	Solubility	Subcellular localization
M	AAY33957.1	Soluble (0.842)	Host cell membrane. Endoplasmic reticulum
N	QBH22433.1	Soluble (0.828)	Cytoplasm
HE	QOV05127.1	Soluble (0.759)	Plasma membrane
S	UZC49500.1	Soluble (0.764)	Plasma membrane
NS2	USW46667.1	Insoluble (0.733)	Cytoplasm
NS12.7	USW46671.1	Insoluble (0.671)	Nucleus (eukaryotic)/interact with DNA (prokaryotic)

*Note*. A solubility index greater than 0.75 after overexpression is defined as the protein is highly soluble.

**Table 3:** Antigenicity, allergenicity, toxicity, and mutability of 13 B-cell epitopes were analyzed.

Proteins	Position	Epitopes	Antigenicity	Allergenicity	Toxicity	Mutability
HE	52–58	PRNYSYM	Yes (1.452)	No	No	No
	294–299	DSRWNN	Yes (1.438)	No	No	No
	318–328	TTNYVGVYDIN	Yes (0.771)	No	No	No

S	341–352	SVPSPLNWERKT	Yes (1.405)	No	No	No
	443–452	FNPSTWNRRF	Yes (0.854)	No	No	No
	618–624	DLQKSNT	Yes (0.624)	No	No	No
	760–770	YSTKRRSRRSI	No (0.499)	No	No	No
	1,200–1,209	TGSGYYYPEP	Yes (0.604)	No	No	No
	1,220–1,231	TCAVNYTKAPDV	Yes (0.626)	No	No	No

M	115–121	WSFNPET	Yes (1.129)	No	No	No
	214–222	STQKGSGLD	Yes (0.934)	No	No	No

N	195–202	APNSRSTS	Yes (1.211)	No	No	No
	269–277	KPRQKRSPN	Yes (0.510)	No	No	No

**Table 4:** Analysis of antigenicity, allergenicity, toxicity, mutability, and immunogenicity of 11 CTL epitopes.

Proteins	Position	Epitopes	Antigenicity	Allergenicity	Toxicity	Mutability	Immunogenicity
HE	137–145	MAVYRSLTF	Yes (0.651)	No	No	No	Yes (0.082)
	364–372	SSVWPLYPY	Yes (0.566)	No	No	No	Yes (0.205)
	408–416	IIIVVLLLY	Yes (0.552)	No	No	No	Yes (0.098)

S	128–136	GSTFVNTSY	Yes (0.656)	No	No	No	Yes (0.100)
	685–693	YSGRVSAAF	Yes (0.898)	No	No	No	Yes (0.003)
	790–798	SLEPVGGLY	Yes (0.657)	No	No	No	Yes (0.115)
	985–993	SAAAGVPFY	Yes (0.523)	No	No	No	Yes (0.182)

M	27–35	FSLGIILLF	Yes (0.970)	No	No	No	Yes (0.269)
	92–100	VAIIMWIVY	No (0.407)	No	No	No	Yes (0.351)

N	72–80	FQKGKEFEF	Yes (1.262)	No	No	No	Yes (0.005)
	323–331	FFFGSRLEL	Yes (0.845)	No	No	No	Yes (0.008)

**Table 5:** Analysis of antigenicity, allergenicity, toxicity, mutability, and immunogenicity of 11 HTL epitopes.

Proteins	Position	Epitopes	Antigenicity	Allergenicity	Toxicity	Mutability	Immunogenicity
HE	76–90	NSIFRSFHFTDFYNY	Yes (0.578)	No	No	No	Yes (0.472)
	355–369	QGVFRYDNVSSVWPL	Yes (0.613)	No	No	No	Yes (0.130)

S	3–17	LILLISLPTAFAVIG	Yes (0.503)	No	No	Yes	Yes (0.271)
	312–324	GYTVQPIADVYRRIP	No (0.490)	No	No	No	Yes (0.326)
	1,185–1,199	PKSGYFVYVNNTWMF	Yes (0.760)	No	No	No	Yes (0.275)
	1,263–1,277	LDYINVTFLDLQDEM	Yes (1.796)	No	No	Yes	Yes (0.376)

M	20–34	KFLKEWNFSLGIILL	Yes (0.694)	No	No	No	Yes (0.292)

N	99–113	GYWYRHNRRSFKTA	Yes (0.662)	No	No	No	Yes (0.050)
	122–136	RWYFYYLGTGPHAK	Yes (1.166)	No	No	No	Yes (0.129)
	323–337	FFFGSRLELAKVQNL	No (0.487)	No	No	No	Yes (0.197)

**Table 6:** The results of the allergenicity, antigenicity, and physicochemical property analysis of the constructed vaccines.

Vaccines	Antigenicity	Instability coefficient	Aliphatic index	Hydropathicity	Half-life (hr)	Molecular weight	Theoretical (pI)	Allergenicity
1	Yes (0.600)	39.81	55.95	−0.546	>10	50,547.85	9.99	No
2	Yes (0.518)	39.81	63.97	−0.55	>10	67,562.85	9.85	No
3	Yes (0.556)	34.61	66.65	−0.356	>10	58,827.13	9.64	No
4	Yes (0.574)	38.17	56.5	−0.55	>10	50,524.75	9.99	No
5	No (0.499)	38.59	64.39	−0.553	>10	67,539.75	9.85	No
6	Yes (0.534)	33.22	67.13	−0.359	>10	58,804.04	9.63	No

*Note*. Antigenicity greater than 0.5 and instability coefficient less than 35 are considered as highly antigenically stable proteins, and hydrophilic mean less than 0 is considered as hydrophilic proteins.

**Table 7:** The docking results of two target proteins.

Protein1	Protein2	Binding energy (kcal/mol)	Contact sites (protein1)	Contact sites (protein2)	Combination type
CY	TLR4	−375.49	LYS-499, TYR-527, ALA-525, LYS-402, TYR-342, and TYR-515	HIS-508, TYR-384, LYS-332, GLU-386, and GLY-136	Salt bridge, hydrogen bond, and hydrophobic interaction
	MHC I	−297.65	A: GLN-254, GLU-253, HIS-190, and HIS-191 B: ARG-96 and LYS-19	LYS-288, LYS-299, TYR-329 GLY-324, and SER-404	Salt bridge, hydrogen bond, and hydrophobic interaction
	MHC II	−294.57	A: VAL-117, VAL-119, SER-19, ASN-78, and GLU-71 B: GLU-52 and ARG-4	THR-345, TYR-342, ASN-348, SER-190, LYS-78, TYR-180, and TYR-355	Salt bridge, hydrogen bond, and hydrophobic interaction

**Table 8:** Protein–protein docking score of CY by HDOCK

Vaccine	Receptor	EVDW	EELE	EGB	ESA	Global energy
CY	TLR4 (3RG1)	−895.465 ± 27.672	−9,859.577 ± 104.444	1,845.827 ± 116.086	−109.261 ± 5.975	−9,018.477 ± 135.313
	MHC I (3PWV)	−697.662 ± 32.278	−6,925.785 ± 118.807	1,740.345 ± 118.472	−81.962 ± 3.754	−5,965.065 ± 131.567
	MHC II (1SEB)	−609.213 ± 30.843	−6,911.545 ± 150.394	1,188.913 ± 130.4	−81.828 ± 4.62	−6,413.673 ± 111.955

*Note*. EVDW, van der Waals energy; EELE, eletrostatic energy; EGB, polar solvation energy; ESA, solvent accessible surface energy.

## Data Availability

All data generated and analyzed during this study are available from the first author upon request.

## Supplementary Materials

Supplementary 1 Table 1: Different combinations of vaccine constructs.

Supplementary 2 Figure 1: The refinement and validation of tertiary structure of three constructions. *Note*. (a) Three tertiary structure models. (b) ERRAT, with the overall quality factor of 89.544. (c) Ramachandran plot analysis showing 69.8% residues in the most favored regions, 23.8% residues in additional allowed regions, 4.0% residues in generously allowed regions, and 2.4% in disallowed regions of protein residues. (d) ProSA-web, with a *Z*-score of −3.6.

Supplementary 3 Table 2: Predicted conformational B-cell epitopes.
