# Supplementary material for: Screening of Bovine Coronavirus Multiepitope Vaccine Candidates: An Immunoinformatics Approach
Source: Transbound Emerg Dis. 2024 Jul 18;2024:5986893. doi: 10.1155/2024/5986893 (PMC12016961; doi:10.1155/2024/5986893)
Supplement: Supplementary 1 — Table 1: Different combinations of vaccine constructs. [file 5986893.f1.pdf]

Table S1 Amino acid sequence information of six multiepitope vaccines

Vaccine construct	Composition	Complete sequence	No. of amino acids
1	Predicted BCL(HE-S-M-N), HTL(HE-S-M-N)&CTL (HE-S-M-N)epitopes with Beta-defensin adjuvant & PADRE sequence	**EAAAK**GIINTLQKYYCRVRGGRCAVLSCLPKEEQIGKCSTRGRKCCRRKK**EAAAK**AKFVAAWTLKAAAKKPRNYSYMKKDSRWNNKKTTNYVGVYDINKKSVPSPLNWERKTKKFNPSTWNRRFKKDLQKSNTKKTGSGYYYPEPKKTCAVNYTKAPDVKKWSFNPETKKSTQKGSGLDKKAPNSRSTSKKKPRQKRSPNGPGPGNSIFRSFHFTDFYNYGPGPGQGVFRYDNVSSVWPLGPGPGPKSGYFVYVNNTWMFGPGPGKFLKEWNFSLGIILLGPGPGGYWYRHNRRSFKTADGPGPGRWYFYYLGTGPHAKDAAYMAVYRSLTFAAYSSVWPLYPYAAYIIIVVLLLYAAYGSTFVNTSYAAYYSGRVSAAFAAYSLEPVGGLYAAYSAAAGVPFYAAYFSLGIILLFAAYFQKGKEFEFAAYFFFGSRLELAAYHHHHHH	449
2	Predicted BCL(HE-S-M-N), HTL(HE-S-M-N)&CTL (HE-S-M-N)epitopes with HABA adjuvant & PADRE sequence	**EAAAK**MAENPNIDDLPAPLLAALGAADLALATVNDLIANLRERAEETRAETRTRVEERRARLTKFQEDLPEQFIELRDKFTTEELRKAAEGYLEAATNRYNELVERGEAALQRLRSQTAFEDASARAEGYVDQAVELTQEALGTVASQTRAVGERAAKLVGIELPGKAEAAGKKAQKAIAKAPAKKASAKKAPAKKAPAKKAAAKKVTQKEAAAKAKFVAAWTLKAAAKKPRNYSYMKKDSRWNNKKTTNYVGVYDINKKSVPSPLNWERKTKKFNPSTWNRRFKKDLQKSNTKKTGSGYYYPEPKKTCAVNYTKAPDVKKWSFNPETKKSTQKGSGLDKKAPNSRSTSKKKPRQKRSPNGPGPGNSIFRSFHFTDFYNYGPGPGQGVFRYDNVSSVWPLGPGPGPKSGYFVYVNNTWMFGPGPGKFLKEWNFSLGIILLGPGPGGYWYRHNRRSFKTADGPGPGRWYFYYLGTGPHAKDAAYMAVYRSLTFAAYSSVWPLYPYAAYIIIVVLLLYAAYGSTFVNTSYAAYYSGRVSAAFAAYSLEPVGGLYAAYSAAAGVPFYAAYFSLGIILLFAAYFQKGKEFEFAAYFFFGSRLELAAYHHHHHH	609
3	Predicted BCL(HE-S-M-N), HTL(HE-S-M-N)&CTL (HE-S-M-N)epitopes with L7/L12 ribosomal protein adjuvant & PADRE sequence	EAAAKMAKLSTDELLDAFKEMTLLELSDFVKKFEETFEVTAAAPVAVAAAGAAPAGAAVEAAEEQSEFDVILEAAGDKKIGVIKVVREIVSGLGLKEAKDLVDGAPKPLLEKVAKEAADEAKAKLEAAGATVTVKEAAAKAKFVAAWTLKAAAKKPRNYSYMKKDSRWNNKKTTNYVGVYDINKKSVPSPLNWERKTKKFNPSTWNRRFKKDLQKSNTKKTGSGYYYPEPKKTCAVNYTKAPDVKKWSFNPETKKSTQKGSGLDKKAPNSRSTSKKKPRQKRSPNGPGPGNSIFRSFHFTDFYNYGPGPGQGVFRYDNVSSVWPLGPGPGPKSGYFVYVNNTWMFGPGPGKFLKEWNFSLGIILLGPGPGGYWYRHNRRSFKTADGPGPGRWYFYYLGTGPHAKDAAYMAVYRSLTFAAYSSVWPLYPYAAYIIIVVLLLYAAYGSTFVNTSYAAYYSGRVSAAFAAYSLEPVGGLYAAYSAAAGVPFYAAYFSLGIILLFAAYFQKGKEFEFAAYFFFGSRLELAAYHHHHHH	534

**4**	Predicted HE(BCL-HTL-CTL), S(BCL-HTL-CTL),M(BCL-HTL-CTL)&N(BCL-HTL-CTL)epitopes with Beta-defensin adjuvant & PADRE sequence	EAAAKGIINTLQKYYCRVRGGRCAVLSCLPKEEQIGKCSTRGRKCCRRKKEAAAKAKFVAAWTLKAAAKKPRNYSYMKKDSRWNNKKTTNYVGVYDINGPGPGNSIFRSFHFTDFYNYGPGPGQGVFRYDNVSSVWPLAAYMAVYRSLTFAAYSSVWPLYPYAAYIIIVVLLLYKKTTNYVGVYDINKKSVPSPLNWERKTKKFNPSTWNRRFKKDLQKSNTKKTGSGYYYPEPGPGPGPKSGYFVYVNNTWMFAAYGSTFVNTSYAAYYSGRVSAAFAAYSLEPVGGLYAAYSAAAGVPFYKKWSFNPETKKSTQKGSGLDGPGPGKFLKEWNFSLGIILLAAYFSLGIILLFKKAPNSRSTSKKKPRQKRSPNGPGPGGYWYRHNRRSFKTADGPGPGRWYFYYLGTGPHAKDAAYFQKGKEFEFAAYFFFGSRLELAAYHHHHHH	448
**5**	Predicted HE(BCL-HTL-CTL), S(BCL-HTL-CTL),M(BCL-HTL-CTL)&N(BCL-HTL-CTL)epitopes with HABA adjuvant & PADRE sequence	EAAAKMAENPNIDDLPAPLLAALGAADLALATVNDLIANLRERAEETRAETRTRVEERRARLTKFQEDLPEQFIELRDKFTTEELRKAAEGYLEAATNRYNELVERGEAALQRLRSQTAFEDASARAEGYVDQAVELTQEALGTVASQTRAVGERAAKLVGIELPGKAEAAGKKAQKAIAKAPAKKASAKKAPAKKAPAKKAAAKKVTQKEAAAKAKFVAAWTLKAAAKKPRNYSYMKKDSRWNNKKTTNYVGVYDINGPGPGNSIFRSFHFTDFYNYGPGPGQGVFRYDNVSSVWPLAAYMAVYRSLTFAAYSSVWPLYPYAAYIIIVVLLLYKKTTNYVGVYDINKKSVPSPLNWERKTKKFNPSTWNRRFKKDLQKSNTKKTGSGYYYPEPGPGPGPKSGYFVYVNNTWMFAAYGSTFVNTSYAAYYSGRVSAAFAAYSLEPVGGLYAAYSAAAGVPFYKKWSFNPETKKSTQKGSGLDGPGPGKFLKEWNFSLGIILLAAYFSLGIILLFKKAPNSRSTSKKKPRQKRSPNGPGPGGYWYRHNRRSFKTADGPGPGRWYFYYLGTGPHAKDAAYFQKGKEFEFAAYFFFGSRLELAAYHHHHHH	608
**6**	Predicted HE(BCL-HTL-CTL), S(BCL-HTL-CTL),M(BCL-HTL-CTL)&N(BCL-HTL-CTL)epitopes with L7/L12 ribosomal adjuvant & PADRE sequence	EAAAKMAKLSTDELLDAFKEMTLLELSDFVKKFEETFEVTAAAPVAVAAAGAAPAGAAVEAAEEQSEFDVILEAAGDKKIGVIKVVREIVSGLGLKEAKDLVDGAPKPLLEKVAKEAADEAKAKLEAAGATVTVKEAAAKAKFVAAWTLKAAAKKPRNYSYMKKDSRWNNKKTTNYVGVYDINGPGPGNSIFRSFHFTDFYNYGPGPGQGVFRYDNVSSVWPLAAYMAVYRSLTFAAYSSVWPLYPYAAYIIIVVLLLYKKTTNYVGVYDINKKSVPSPLNWERKTKKFNPSTWNRRFKKDLQKSNTKKTGSGYYYPEPGPGPGPKSGYFVYVNNTWMFAAYGSTFVNTSYAAYYSGRVSAAFAAYSLEPVGGLYAAYSAAAGVPFYKKWSFNPETKKSTQKGSGLDGPGPGKFLKEWNFSLGIILLAAYFSLGIILLFKKAPNSRSTSKKKPRQKRSPNGPGPGGYWYRHNRRSFKTADGPGPGRWYFYYLGTGPHAKDAAYFQKGKEFEFAAYFFFGSRLELAAYHHHHHH	533
