# Supplementary figures and images for: Screening of Bovine Coronavirus Multiepitope Vaccine Candidates: An Immunoinformatics Approach
Source: Transbound Emerg Dis. 2024 Jul 18;2024:5986893. doi: 10.1155/2024/5986893 (PMC12016961; doi:10.1155/2024/5986893)

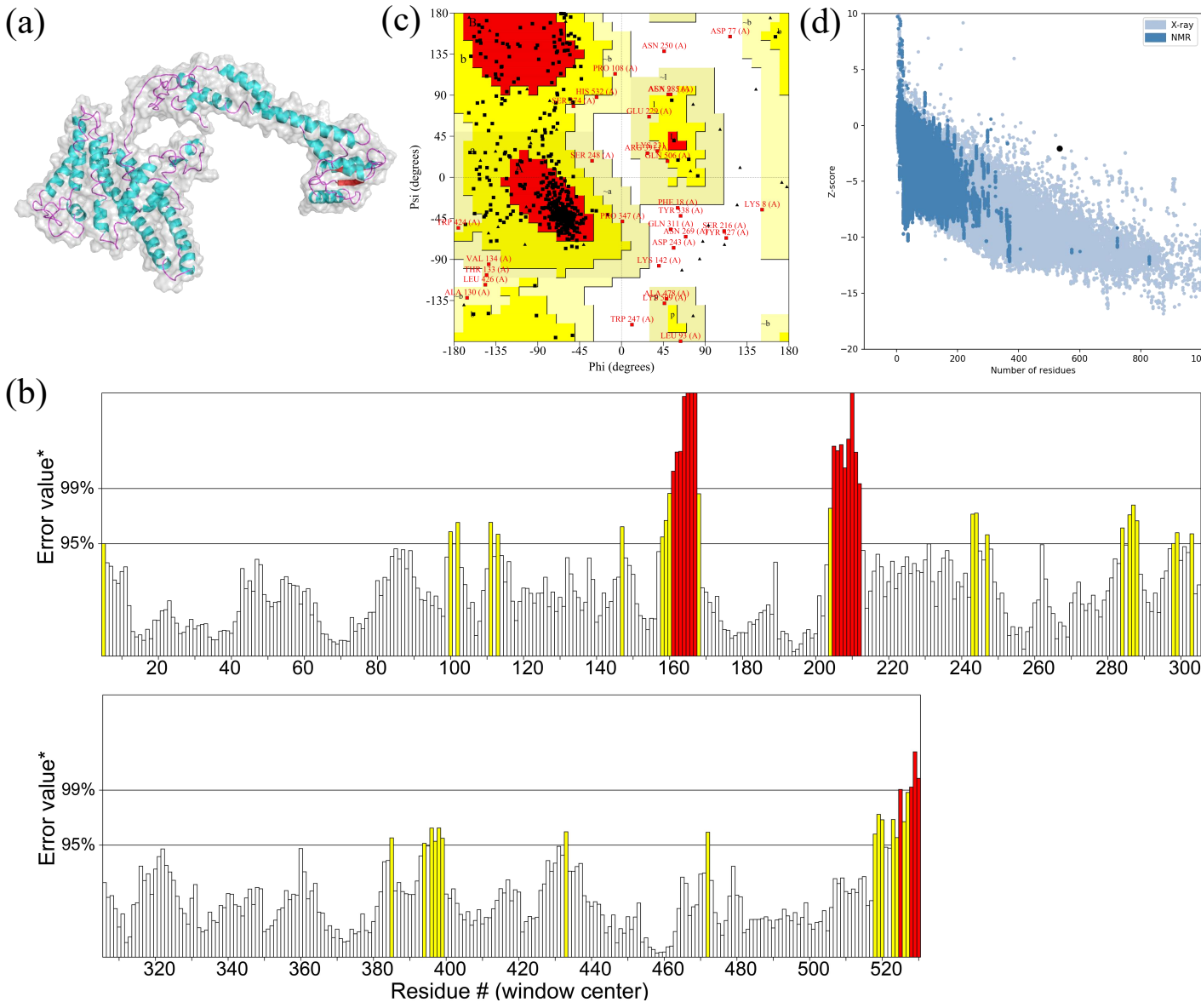

Supplement: Supplementary 2 — Figure 1: The refinement and validation of tertiary structure of three constructions. Note. (a) Three tertiary structure models. (b) ERRAT, with the overall quality factor of 89.544. (c) Ramachandran plot analysis showing 69.8% residues in the most favored regions, 23.8% residues in additional allowed regions, 4.0% residues in generously allowed regions, and 2.4% in disallowed regions of protein residues. (d) ProSA-web, with a Z-score of −3.6. [file 5986893.f2.pdf]
