# Supplementary material for: Screening of Bovine Coronavirus Multiepitope Vaccine Candidates: An Immunoinformatics Approach
Source: Transbound Emerg Dis. 2024 Jul 18;2024:5986893. doi: 10.1155/2024/5986893 (PMC12016961; doi:10.1155/2024/5986893)
Supplement: Supplementary 3 — Table 2: Predicted conformational B-cell epitopes. [file 5986893.f3.pdf]

Table S2 Predicted conformational B cell epitopes

Serial numb	Residues	Number of residues	Score
A	A: F476, A: K477, A: T478, A: A479, A: D480, A: G481, A: P482, A: G483, A: P484, A: G485, A: R486, A: W487, A: Y488, A: F489	14	0.789
B	A: G403, A: S404, A: G405, A: L406, A: D407, A: G408, A: P409, A: G410, A: P411, A: G412, A: K413, A: F414, A: L415, A: K416, A: E417, A: N419, A: F420, A: L422, A: G423, A: I436, A: L437, A: L438, A: F439, A: K440, A: K441, A: A442, A: P443, A: N444, A: S445, A: R446, A: S447, A: T448, A: S449, A: K450, A: K451, A: R457, A: S458, A: P459, A: N460, A: G461, A: P462, A: G463, A: P464, A: G465, A: G466, A: Y467, A: Y469, A: R470, A: Y491, A: L492, A: G493, A: T494, A: G495, A: P496, A: H497, A: A498, A: D500, A: A501, A: A502, A: Y503, A: F504, A: Q505, A: K506, A: G507, A: K508, A: E509, A: F510, A: F518, A: G519, A: S520, A: R521, A: L522, A: E523, A: L524, A: A525, A: A526, A: Y527, A: H528, A: H529, A: H531, A: H532, A: H533	82	0.711
C	A: E1, A: A2, A: A3, A: A4, A: K5, A: M6, A: A7, A: K8, A: L9, A: M21, A: P54, A: A55, A: G56, A: A57, A: A58, A: V59, A: E60, A: A61, A: A62, A: E63, A: E64, A: Q65, A: S66, A: E67, A: F68, A: D69, A: V70, A: I71, A: L72, A: E73, A: A74, A: A130, A: T131, A: V132, A: T133, A: V134, A: K135, A: E136, A: A137, A: A138, A: A139, A: K140, A: A141, A: K142, A: F143, A: T148, A: A151, A: A152, A: K155, A: P156, A: N158, A: Y159, A: S160, A: Y161, A: M162, A: K164, A: D165, A: S166, A: R167, A: W168, A: N169, A: N170, A: K171, A: K172, A: T173, A: T174, A: N175, A: Y176, A: V177, A: G178, A: V179, A: Y180, A: D181, A: I182, A: N183, A: G184, A: P185, A: D270, A: I271, A: N272, A: K273, A: S275, A: V276, A: P277, A: S278, A: P279, A: L280, A: N281, A: W282, A: E283, A: T286, A: K287	92	0.711
D	A: K96, A: A98, A: K99, A: D100, A: L101, A: V102, A: D103, A: G104, A: A105, A: P106, A: K107, A: P108, A: L109, A: L110, A: E111, A: K112, A: V113, A: A114, A: K115, A: E116, A: D119, A: K163, A: S218, A: Y226, A: V229, A: R231, A: S232, A: L233, A: T234, A: F235, A: A236, A: A237, A: Y238, A: S239, A: S240, A: W242, A: P243, A: L244, A: Y245, A: P246, A: Y247, A: A248	42	0.685

E	A: F298, A: K299, A: L302, A: Q303, A: K304, A: S305, A: N306, A: T307, A: K308, A: T310, A: S312, A: G313, A: Y314, A: Y315, A: Y316, A: P317, A: E318, A: P319, A: G320, A: P321, A: G322, A: P323, A: G324, A: P325, A: K326, A: S327, A: G328, A: Y329, A: Y387, A: K388, A: K389, A: W390, A: S391, A: F392, A: N393, A: E395, A: K397, A: K398, A: Q455, A: K456	40	0.666
